# Electron donor concentration- and pH-dependent, biogenic Fe(II)-facilitated biotransformation of ferrihydrite to various iron oxide nanomaterials by *Shewanella* sp. strain HN-41

**DOI:** 10.1128/aem.00717-26

**Published:** 2026-05-20

**Authors:** Yongseok Ko, Hor-Gil Hur, Ji-Hoon Lee

**Affiliations:** 1 School of Earth Sciences and Environmental Engineering, Gwangju Institute of Science and Technology (GIST) 542529, Gwangju, Republic of Korea; 2 Department of Bioenvironmental Chemistry, Jeonbuk National University 26714, Jeonju, Republic of Korea; Michigan State University, East Lansing, Michigan, USA

**Keywords:** biotransformation, iron oxide nanomaterials, ferrihydrite, hematite, goethite, magnetite, lepidocrocite, *Shewanella*sp. HN-41

## Abstract

Dissimilatory iron-reducing bacteria catalyze the biotransformation of ferrihydrite into iron oxide nanomaterials by producing aqueous Fe(II), which subsequently transfers electrons to ferrihydrite. However, the specific environmental factors that determine the biotransformation of ferrihydrite to distinct iron oxide nanomaterials remain poorly understood. In this study, we investigated the biotransformation of ferrihydrite to various iron oxide nanomaterials using the iron-reducing bacterium *Shewanella* sp. strain HN-41, depending on lactate concentrations from 0.1 to 10 mM at pH 6.8 and 7.5. SEM and XRD analyses revealed that lepidocrocite nanosheets were generated at 0.1–1 mM lactate and pH 6.8. In comparison, at pH 7.5, secondary clusters of hematite nanoparticles and nanorod clusters of goethite were generated at 0.1 mM and 1 mM lactate, respectively. However, at a higher concentration of 10 mM lactate, magnetite nanoparticles, and nanorods were generated, regardless of pH. Strain HN-41 produced higher aqueous Fe(II) concentrations and lowered Eh values as lactate concentration increased. In addition, overall aqueous Fe(II) concentrations and Eh values were higher at pH 6.8 than at pH 7.5. The differences in aqueous Fe(II) concentration and the system’s redox potential (Eh value) altered the fate of aqueous Fe(II), resulting in the biotransformation products of various iron oxide nanomaterials. This study provides insight into how nutrient availability and pH affect the geochemical composition of iron minerals by regulating the activity of iron-reducing bacteria.

**IMPORTANCE:**

Ferrihydrite is ubiquitous in the environment and a favorable electron acceptor for iron-reducing bacteria due to its low crystallinity and high surface area. After reduction of ferrihydrite by the iron-reducing bacteria, it was transformed into thermodynamically more stable crystalline iron oxide nanomaterials by electron transfer from biogenic aqueous Fe(II) to ferrihydrite. This study highlighted that electron donor concentration and pH conditions strongly influenced the biotransformation products of distinct iron oxide nanomaterials by affecting the activity of dissimilatory iron-reducing bacteria.

## INTRODUCTION

Ferrihydrite is a low-crystalline and nanoparticulated Fe(III) oxyhydroxide commonly found in soil, sediments, and groundwater aquifers (1). Owing to its intrinsic instability, ferrihydrite is spontaneously transformed into thermodynamically more stable crystalline iron oxides, but slowly in the absence of catalysts (2). In anoxic environments, ferrihydrite is considered a favorable source of Fe(III) as an electron acceptor for dissimilatory iron-reducing bacteria due to its low crystallinity and high surface area (2, 3). Dissimilatory iron-reducing bacteria play key roles in the transformation of ferrihydrite into various iron oxide nanomaterials by reducing Fe(III) in ferrihydrite to aqueous Fe(II), which in turn catalyzes the electron transfer from aqueous Fe(II) to ferrihydrite (2–5). The types of iron oxides transformed from ferrihydrite were governed by the environmental conditions surrounding ferrihydrite, such as aqueous Fe(II) concentration, temperature, pH, and reduction potential (Eh) (2, 6–9). Interestingly, several studies using *Shewanella oneidensis* MR-1 mutants in iron-reducing systems or varying electron donor concentrations have demonstrated that dissimilatory iron-reducing bacteria could govern ferrihydrite biotransformation into mixed phases of various iron oxides by adjusting aqueous Fe(II) production and Eh values (3, 10, 11). However, the specific factors and conditions governing the biotransformation of the distinct iron oxide nanomaterials have not been sufficiently investigated compared to abiotic ferrihydrite transformation. Considering the ubiquity and roles of dissimilatory iron-reducing bacteria, understanding the dynamics of biotransformation is important for elucidating the geochemical cycling of iron minerals. The objective of this study is to understand the conditions for the biotransformation of ferrihydrite to distinct iron oxide nanomaterials by the dissimilatory iron-reducing bacterium *Shewanella* sp. HN-41 depends on electron donor concentration and pH.

## RESULTS AND DISCUSSION

### Biotransformation of iron oxide nanomaterials depending on lactate concentration and pH

*Shewanella* sp. strain HN-41 transformed poorly crystalline ferrihydrite into various iron oxide nanomaterials by reducing ferrihydrite, depending on pH and lactate concentration. The biotransformation of ferrihydrite is an important process in the environment because the transformed iron oxides are more persistent than ferrihydrite and can modify the soil properties by altering surface reactivity, redox properties, and sorption of organic and inorganic matter (1, 12, 13).

**Fig 1:**
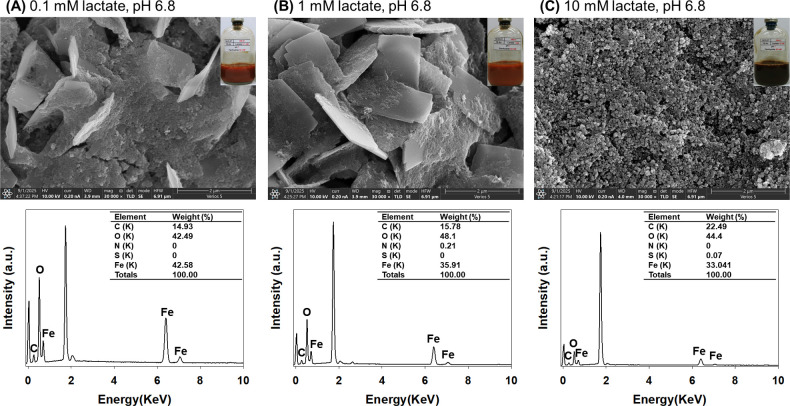
SEM images and EDS spectra of biogenic iron oxide nanomaterials formed by *Shewanella* sp. HN-41 at pH 6.8 under different lactate concentrations: 0.1 mM (**A**, lepidocrocite), 1 mM (**B**, lepidocrocite), and 10 mM (**C**, magnetite).

**Fig 2:**
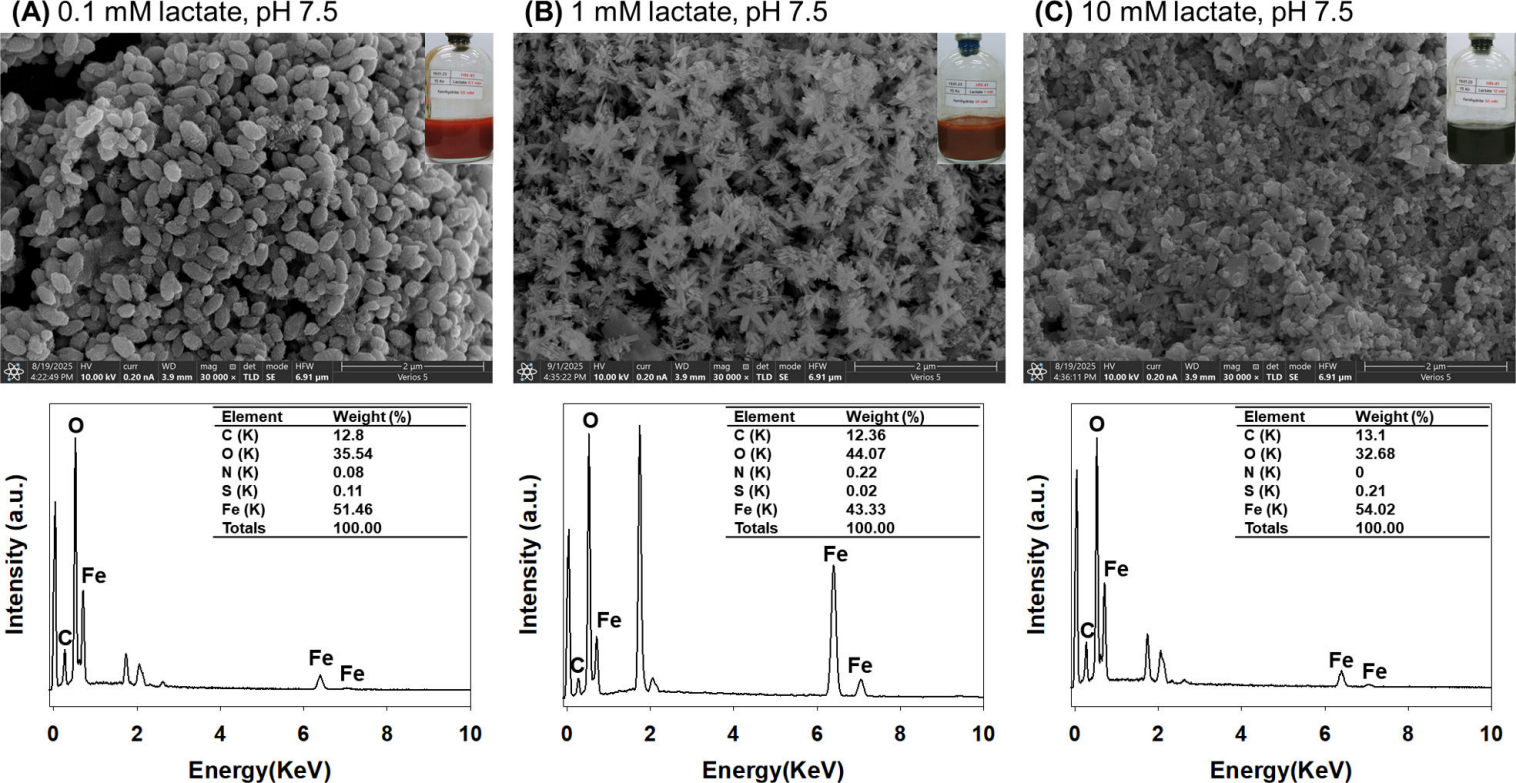
SEM images and EDS spectra of biogenic iron oxide nanomaterials formed by *Shewanella* sp. HN-41 at pH 7.5 under different lactate concentrations: 0.1 mM (**A**, hematite), 1 mM (**B**, goethite), and 10 mM (**C**, magnetite).

After 16 d of incubation with strain HN-41, the dark brown color of ferrihydrite changed to red-orange, brown, and dark colors of iron (hydr)oxides at lactate concentrations of 0.1 mM, 1 mM, and 10 mM, respectively, at both pH 6.8 and 7.5 (Fig. 1 and 2). SEM analysis showed that ferrihydrite exhibited a morphology of uniformly dispersed nanoparticles (Fig. S1), which were transformed into nanosheets at lactate concentrations of 0.1 mM and 1 mM at pH of 6.8 (Fig. 1A and B). Quite different morphologies of the transformed iron (hydr)oxides were observed at pH 7.5. Ferrihydrite was transformed into secondary clusters of nanoparticles under 0.1 mM lactate and nanorod clusters under 1 mM (Fig. 2A and B). In comparison, at a higher concentration of 10 mM lactate, discrete smaller nanoparticles or a mixture of nanoparticles and nanorods were generated at pH 6.8 and 7.5 (Fig. 1C and 2C). Energy-dispersive X-ray spectroscopy (EDS) analysis showed that the nanomaterials consisted mainly of iron and oxygen, indicating that the observed nanomaterials were iron oxides (Fig. 1 and 2).

**Fig 3:**
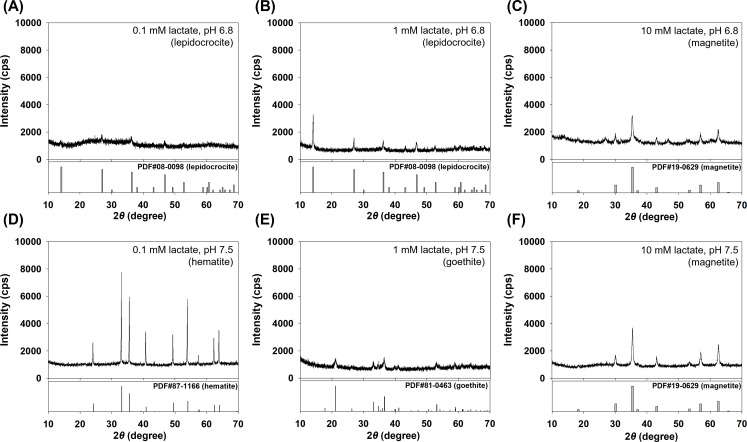
XRD spectra of biogenic iron oxide nanomaterials formed by *Shewanella* sp. HN-41 under different lactate concentrations: 0.1 mM (**A**, lepidocrocite), 1 mM (**B**, lepidocrocite), 10 mM (**C**, magnetite) at pH 6.8 and 0.1 mM (**D**, hematite), 1 mM (**E**, goethite), and 10 mM (**F**, magnetite) at pH 7.5.

The iron oxide nanomaterials were characterized using XRD analysis through comparison of major peaks with the reference mineral database: Powder Diffraction File (PDF) numbers 08-0098 (lepidocrocite, *γ*-Fe(III)O(OH)), 87-1166 (hematite, *α*-Fe(III)_2_O_3_), 81-0463 (goethite, *α*-Fe(III)O(OH)), and 19-0629 (magnetite, Fe(II)Fe(III)_2_O_4_). The iron oxide nanosheets formed at pH 6.8 in the presence of 0.1 mM and 1 mM lactate at pH 6.8 were identified as lepidocrocite based on PDF 08-0098 (lepidocrocite) (Fig. 3A and B). However, the overall intensity of XRD peaks related to lepidocrocite (2*θ* = 14, 27, 36, and 46°) formed in the presence of 1 mM lactate was significantly higher than that formed in the presence of 0.1 mM lactate. The higher XRD peak intensity observed under the 1 mM lactate conditions is attributed to the formation of a more crystalline phase of iron oxide nanosheets (lepidocrocite), but a less abundant amorphous phase of nanoparticles (ferrihydrite) compared to that under 0.1 mM lactate conditions (Fig. 1A and B). At pH 7.5, secondary clusters of iron oxide nanoparticles formed in the presence of 0.1 mM lactate were identified as hematite, consistent with PDF 87-1166 (hematite, *α*-Fe(III)_2_O_3_), while iron oxide nanorod clusters formed in the presence of 1 mM lactate were identified as goethite based on PDF 81-0463 (goethite, *α*-Fe(III)O(OH)) (Fig. 3D and E). The discrete nanoparticles and nanorods transformed under 10 mM lactate at both pH 6.8 and 7.5 were consistent with PDF 19-0629 (magnetite, Fe(II)Fe(III)_2_O_4_) (Fig. 3C and F). However, XRD analysis was not sufficient to identify magnetite owing to peak similarity with other iron oxides, such as maghemite. Nevertheless, we characterized the discrete nanoparticles and nanorods as magnetite because the dark-colored precipitates consisting of discrete nanoparticles and nanorods transformed by iron-reducing bacteria or chemical synthesis under relatively high Fe(II) conditions have been characterized as magnetite (4, 12, 14, 15). The morphology and XRD patterns of ferrihydrite remained unchanged in all control conditions without strain HN-41 after 16 d of incubation, confirming that the transformation of iron oxide nanomaterials occurred through the biological activity of strain HN-41 (Fig. S2 and S3).

### Changes in lactate, aqueous Fe(II), pH, and reduction potential during biotransformation of iron oxide nanomaterials

**Fig 4:**
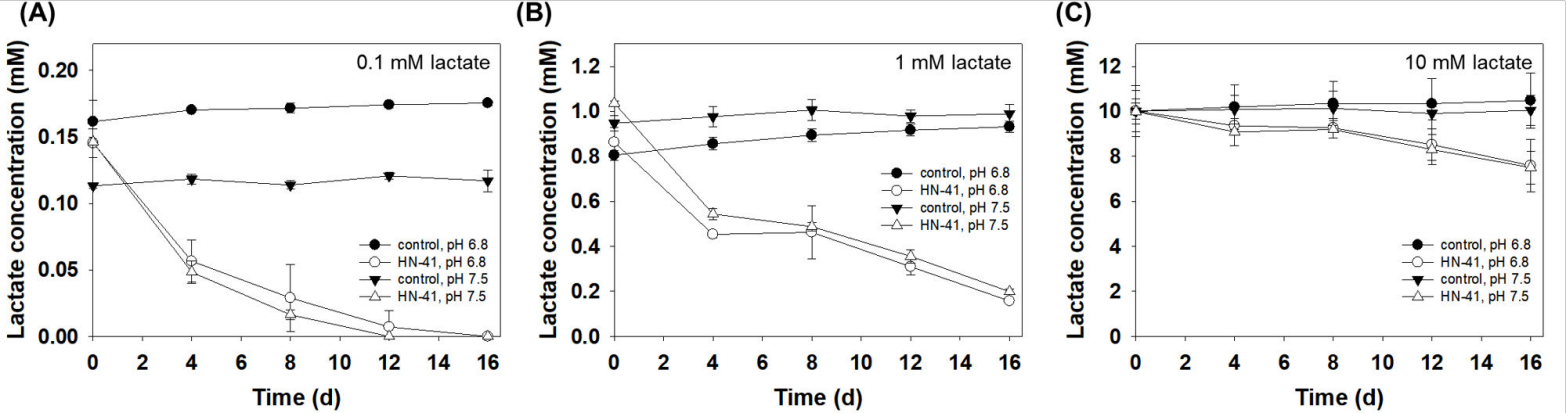
Lactate concentrations changed during the formation of iron oxide nanomaterials by *Shewanella* sp. HN-41 under different incubation conditions: 0.1 mM lactate (**A**), 1 mM lactate (**B**), and 10 mM lactate (**C**).

**Fig 5:**
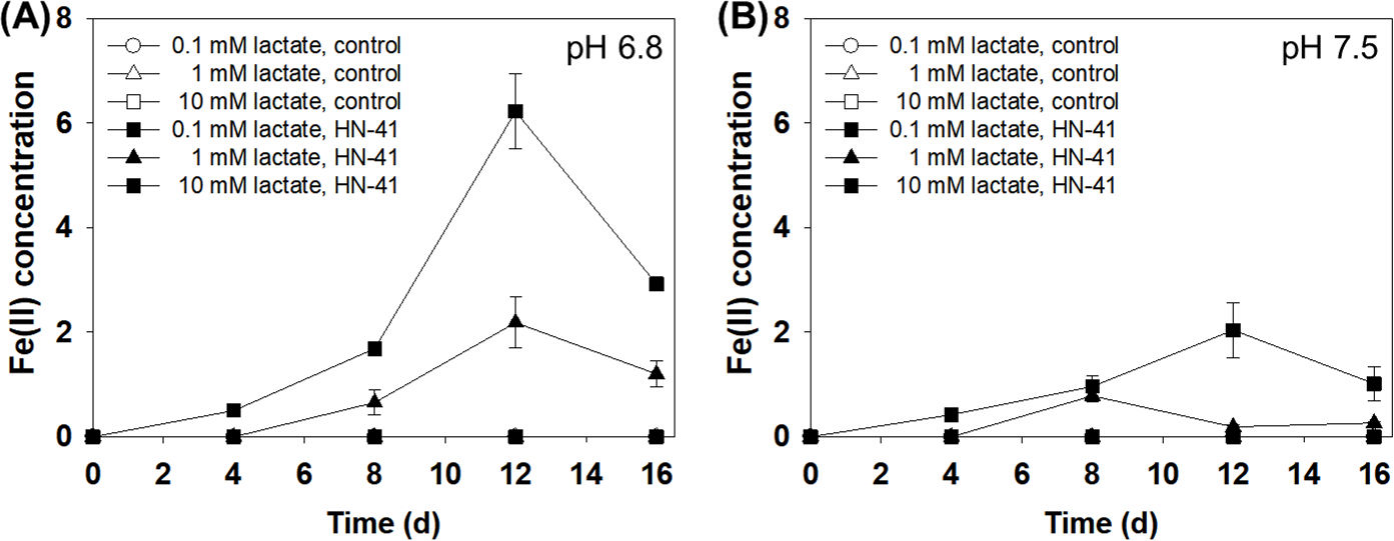
Fe(II) concentrations changed during the formation of iron oxide nanomaterials by *Shewanella* sp. HN-41 under different incubation conditions at pH 6.8 (**A**) and 7.5 (**B**).

During iron oxide nanomaterial biotransformation by strain HN-41, lactate was consumed as a sole electron donor coupled to the dissimilatory reduction of Fe(III) in ferrihydrite to aqueous Fe(II) as an electron acceptor (15). After 16 d of incubation, lactate consumption was approximately 0.1 mM, 0.75 mM, and 2.5 mM under 0.1 mM, 1 mM, and 10 mM lactate conditions, respectively, showing insignificant differences in the electron donor consumption patterns between pH 6.8 and 7.5 (Fig. 4). However, the released maximum aqueous Fe(II) concentrations were approximately three times higher at pH 6.8 (6.2 mM and 2.2 mM under 1 mM and 10 mM lactate, respectively) than at pH 7.5 (2.0 mM and 0.8 mM, respectively) (Fig. 5). The released aqueous Fe(II) concentrations decreased to approximately half of their maximum values under all lactate conditions at the end of incubation, indicating that the aqueous Fe(II) was consumed during biotransformation. into iron oxide nanomaterials. According to the known stoichiometry, four Fe(III) ions are reduced to Fe(II) by the oxidation of one lactate molecule to CO_2_ and acetate (16, 17). However, the measured aqueous Fe(II) concentrations were significantly lower than the stoichiometrically estimated Fe(II) concentration (e.g., ~10 mM Fe(II) estimated from 2.5 mM lactate consumption). This discrepancy suggests that a substantial fraction of the biogenic Fe(II) was sequestered from the aqueous phase, likely through participation in the biotransformation of ferrihydrite. Consequently, the measured aqueous Fe(II) concentrations underestimate the total Fe(II) production by strain HN-41. Hence, trace amounts of aqueous Fe(II) were speculated to be produced during the consumption of 0.1 mM of lactate and promptly consumed for the biotransformation into hematite and lepidocrocite. Lactate consumption and release of aqueous Fe(II) were not observed under all control conditions without strain HN-41 after 16 d of incubation (Fig. 4 and 5).

The pH and Eh values of the culture medium decreased during the biotransformation of iron oxide nanomaterials by strain HN-41 (Tables 1 and 2). The pH of the culture medium decreased slightly after 16 d of incubation regardless of lactate concentration, from 6.8 and 7.5 to 6.7 and 7.3, respectively. In contrast, Eh values substantially decreased after 16 d of incubation to ranges of −61 to −236 mV at pH 6.8 and −154 to −289 mV at pH 7.5 as the lactate concentration increased from 0.1 mM to 10 mM. The overall Eh values were significantly lower at pH 7.5 than at pH 6.8 under all lactate conditions throughout the incubation periods.

**TABLE 1:** Changes in pH and Eh during biogenic iron oxide nanomaterial formation by *Shewanella* sp. HN-41 at pH 6.8 under different lactate concentrations

Time (d)	0.1 mM lactate, HN-41		1 mM lactate, HN-41		10 mM lactate, HN-41	
	pH	Eh (mV)	pH	Eh (mV)	pH	Eh (mV)
0	6.8	165.5 ± 3.5	6.8	156.5 ± 1.5	6.8	154.5 ± 2.5
8	6.7	−47 ± 5	6.7	−106 ± 2	6.7	−159.5 ± 24.5
16	6.7	−61.5 ± 12.5	6.7	−178 ± 5	6.7	−236.5 ± 3.5

**TABLE 2:** Changes in pH and Eh during biogenic iron oxide nanomaterial formation by *Shewanella* sp. HN-41 at pH 7.5 under different lactate concentrations

Time (d)	0.1 mM lactate, HN-41		1 mM lactate, HN-41		10 mM lactate, HN-41	
	pH	Eh (mV)	pH	Eh (mV)	pH	Eh (mV)
0	7.5	151 ± 1	7.5	176 ± 1	7.5	163 ± 2
8	7.3	−117 ± 13	7.3	−148 ± 31	7.4	−193.5 ± 50
16	7.2	−154.5 ± 18	7.3	−271.5 ± 10	7.3	−289 ± 1

### Mechanism of various iron oxide nanomaterial biotransformation

The primary mechanism of ferrihydrite transformation is the electron transfer of Fe(II) on the surface of ferrihydrite to structural Fe(III), facilitating the formation of thermodynamically more stable crystalline iron (hydr)oxides, including magnetite, goethite, lepidocrocite, and hematite (1, 6, 9, 18, 19). The types of formed iron oxides can be controlled by adjusting chemical reaction conditions such as aqueous Fe(II) concentrations, temperature, pH, and Eh values (2, 6–9). For instance, magnetite was produced under relatively high aqueous Fe(II) concentrations, while goethite, lepidocrocite, and hematite formed under relatively low aqueous Fe(II) concentrations upon controlling reaction conditions of temperature, pH, and Eh (6, 7, 14).

The biotransformation of ferrihydrite to iron oxide nanomaterials by strain HN-41 was inferred to be similarly influenced by pH, aqueous Fe(II) concentrations, and Eh values adjusted by the rate of dissimilatory iron reduction, depending on lactate concentrations (3, 10, 11). Under 10 mM lactate conditions at both pH 6.8 and 7.5, strain HN-41 produced high aqueous Fe(II) concentrations and low Eh values, conditions that presumably promoted the formation of magnetite, a mixed-valence (Fe(II)Fe(III)_2_) iron oxide that typically forms under highly reduced, Fe(II)-rich conditions (10, 11, 14).

Interestingly, the types of transformed iron oxides varied under relatively low lactate concentrations, depending on its concentration and pH. We assumed that aqueous Fe(II) productions by strain HN-41 were similar at the initial lactate concentrations at both pH 6.8 and 7.5, considering that lactate consumption patterns were similar (Fig. 4). However, aqueous Fe(II) concentrations were consistently higher at pH 6.8 than at pH 7.5 throughout the incubation, likely due to the different fate of aqueous Fe(II) depending on pH and Eh conditions (Fig. 5). The different fate of aqueous Fe(II) was considered to play an important role in the biotransformation of ferrihydrite, yielding various types of iron oxide nanomaterials.

For instance, Boland et al. (18) reported that the Fe(II)-ferrihydrite surface interaction rate at different pH conditions influenced the transformation of ferrihydrite into lepidocrocite and goethite. Specifically, lepidocrocite was transformed under the low Fe(II)-ferrihydrite surface interaction conditions at pH 6.17–6.92, whereas goethite was transformed under the high surface interaction conditions at pH 7.26. Accordingly, ferrihydrite was transformed by strain HN-41 into lepidocrocite under 0.1 mM and 1 mM lactate conditions at pH 6.8, whereas goethite was transformed under 1 mM lactate conditions at pH 7.5. Although interfacial Fe(II)–ferrihydrite interactions were not directly measured in this study, the observed trends correlated with the previous report, indicating that the surface interaction rate influenced the biotransformation of ferrihydrite into lepidocrocite and goethite. To our knowledge, no chemical transformation study has yet been reported that shows a correlation between aqueous Fe(II) concentrations and the formation of goethite and hematite from ferrihydrite. Nevertheless, our results suggest that hematite transformation was favored under lower aqueous Fe(II) concentrations and less reduced conditions compared to the conditions for goethite and magnetite transformation.

This study highlights that electron donor concentrations and pH play an important role in the biotransformation of ferrihydrite to iron oxide nanomaterials by adjusting the production and fate of aqueous Fe(II) produced by dissimilatory iron-reducing bacteria. This finding provides the insight that nutrient and pH conditions of environments could substantially alter the mineralogical composition and geochemical behaviors of iron-bearing materials by influencing the activity of dissimilatory iron-reducing bacteria. Such processes may be particularly relevant in soils, sediments, and subsurface environments, where microbial iron reduction commonly occurs and strongly influences iron cycling.

## MATERIALS AND METHODS

### Chemicals and media

*N*-2-Hydroxyethylpiperazine-*N*′−2-ethanesulfonic acid (HEPES) was purchased from GoldenBio (St. Louis, MO). FeCl_3_·6H_2_O, NaOH, NaHCO_3_, NaCl, NH_4_Cl, *β*-glycerophosphoric acid disodium salt, ferrozine (3-(2-pyridyl)−5,6-diphenyl-1,2,4-triazine-4′,4′′-disulfonic acid sodium salt), CaCl_2_, HCl, and KCl were purchased from Sigma-Aldrich (St. Louis, MO). Yeast extract was purchased from Condalab (Madrid, Spain). Luria-Bertani (LB) broth was purchased from BD Difco (Detroit, MI). Sodium lactate (50%) was purchased from Junsei (Tokyo, Japan). Ferrous ethylene diammonium sulfate tetrahydrate was purchased from Fluka (Buchs, Switzerland).

HEPES-buffered basal medium, named *Shewanella* basal medium, was prepared following Lee et al. (15): HEPES 7.2 g/L, NaHCO_3_ 2.5 g/L, NaCl 7.2 g/L, NH_4_Cl 1 g/L, *β*-glycerophosphoric acid disodium salt 0.06 g/L, CaCl_2_ 0.06 g/L, KCl 0.1 g/L, and yeast extract 0.1 g/L. The pH of the medium was adjusted to pH 7.0 and 7.8, respectively, using 10 M NaOH and 10 M HCl after autoclaving and adding 50 mM ferrihydrite. To prepare anoxic *Shewanella* basal medium, the medium was boiled for 20 min to eliminate dissolved gases, and N_2_ gas (99.9%) was extensively purged into the medium for 30 min. Afterward, the anoxic medium was distributed into serum bottles capped with butyl rubber stoppers and aluminum seals.

Poorly crystalline ferrihydrite was prepared following the modified method of Schwertmann and Cornell et al*.* (20). Briefly, 0.4 M FeCl_3_·6H_2_O was dissolved in distilled water (DW), and pH was carefully increased to pH 6 using 10 M NaOH, resulting in precipitation of dark brown-colored ferrihydrite. The solution was incubated for 12 h at room temperature and 200 rpm. Ferrihydrite was washed three times using anoxic DW by centrifugation at 3,075 *× g* for 10 min at 4℃ and resuspended in DW to a final concentration of 0.55 M under N_2_ gas (99.9%) purging conditions. The prepared ferrihydrite showed poorly crystalline nanoparticles, as shown in Fig. S1. All experiments were performed in an anaerobic glove box (Coy Laboratory, Grass Lake, MI) filled with a mix of gases (N_2_:H_2_:CO_2_ = 90:5:5).

### Biotransformation of iron oxide nanomaterials using *Shewanella* sp. HN-41

Strain HN-41 was incubated in LB broth for 18 h at 30℃ and 180 rpm. The cells were washed three times using 30 mM HEPES buffer (pH 7.0) by centrifugation at 3,075 × *g* for 5 min at 4℃. The washed cells were inoculated into the serum bottles containing *Shewanella* basal media (pH 7.0 and 7.8) at a final OD_600_ = 0.01. Ferrihydrite was supplemented at a final concentration of 50 mM. Lactate was supplemented to serum bottles at a final concentration of 0.1 mM, 1 mM, and 10 mM, respectively. As a control, anoxic DW was added instead of strain HN-41. All serum bottles were incubated at 30℃ under static and dark conditions.

### Measurement of pH, reduction potential, aqueous Fe(II), and lactate

Triplicate serum bottles were sacrificed every 8 d of incubation to measure pH and Eh values of the culture media. The pH and Eh values were measured using HN-31P portable pH/ORP meter (DKK-TOA, Tokyo, Japan) equipped with PST-2729C ORP electrode (DKK-TOA) and a GST-2729C pH electrode (DKK-TOA). The ORP electrode performance was periodically verified using ORP Check Solution (DKK-TOA) following the product instructions. To measure aqueous Fe(II) and lactate concentrations, 0.5 mL aliquots were withdrawn using a syringe. The sample was first filtered through a 0.22 μm polyvinylidene fluoride (PVDF) syringe filter (Whatman-GE Health-cares, Pittsburgh, PA) to remove particulate and solid-associated phases. A 5 μL portion of the filtrate was then transferred to 1 mL of ferrozine solution in 30 mM HEPES buffer (pH 7.0) and measured for absorbance at 562 nm following the ferrozine assay (21). In this study, “aqueous Fe(II)” refers to Fe(II) present in the filtrate through 0.22 μm, which mainly represents dissolved Fe(II) and potentially a small fraction of colloidal Fe species, but excludes Fe(II) associated with particulate and/or solid phases. Therefore, the measured aqueous Fe(II) represents only a subset of the total Fe(II) generated under these conditions, as Fe(II) may also be incorporated into newly formed or existing solid phases. A standard curve of aqueous Fe(II) was prepared using ferrous ethylene diammonium sulfate tetrahydrate dissolved in 30 mM HEPES buffer (pH 7.0).

The remaining aliquots were centrifuged at 1,300 rpm for 1 min. Subsequently, the supernatants were filtered using a 0.22 μm PVDF syringe filter. Lactate concentration was quantified using high-performance liquid chromatography (HPLC, Agilent, Santa Clara, CA) equipped with a Rezex ROA-organic acid H+ (8%) ion exclusion HPLC column (7.8 × 300 mm, 1.8 μm) and a SPD-10A photodiode array detector (Agilent, Santa Clara, CA). The injection volume of the sample was 20 μL. The mobile phase was 5 mM sulfuric acid in DW at a flow rate of 0.5 mL/min for 30 min and a column temperature at 40℃. Lactate was detected at 210 nm.

### Characterization of biogenic iron oxide nanomaterials

Biogenic iron oxide nanomaterials after 16 d of incubation were washed using anoxic DW by centrifugation at 3,075 × g for 5 min at 4℃. The washed samples were mounted on silica wafers and dried in a vacuum desiccator containing silica gel. For SEM analysis, the wafers were mounted onto SEM stubs using carbon tape and subsequently coated with Pt to a thickness of 20 nm using an ion sputter coater (DSR, VAC COAT, London, England). SEM analysis was performed using Veros 5 XHNR SEM (Thermo Scientific, MA, USA) equipped with Ultim Max 65 EDS (Oxford Instruments, Abingdon, England). For XRD analysis, the washed iron oxide nanomaterials were lyophilized at −80℃ under 5 mTorr vacuum for 5 d using a TFD8501 freeze-dryer (Ilshinbiobase, Yangju, South Korea). The samples were analyzed using a SmartLab X-ray diffractometer (Rigaku, Tokyo, Japan) with a scan range of 2*θ* = 10–70° at a scan speed of 5°/min.

## Data Availability

The original contributions presented in the study are included in the article and its supplemental material.

## SUPPLEMENTAL MATERIAL

Supplemental figures aem.00717-26-s0001.docx Figures S1 to S3.

ASM does not own the copyrights to Supplemental Material that may be linked to, or accessed through, an article. The authors have granted ASM a non-exclusive, world-wide license to publish the Supplemental Material files. Please contact the corresponding author directly for reuse.
