# Supplementary material for: Electron donor concentration- and pH-dependent, biogenic Fe(II)-facilitated biotransformation of ferrihydrite to various iron oxide nanomaterials by Shewanella sp. strain HN-41
Source: Appl Environ Microbiol. 2026 May 20;92(6):e00717-26. doi: 10.1128/aem.00717-26 (PMC13274382; doi:10.1128/aem.00717-26)
Supplement: Supplemental figures — Figures S1 to S3. [file aem.00717-26-s0001.docx]

**[Supplementary materials]**

**
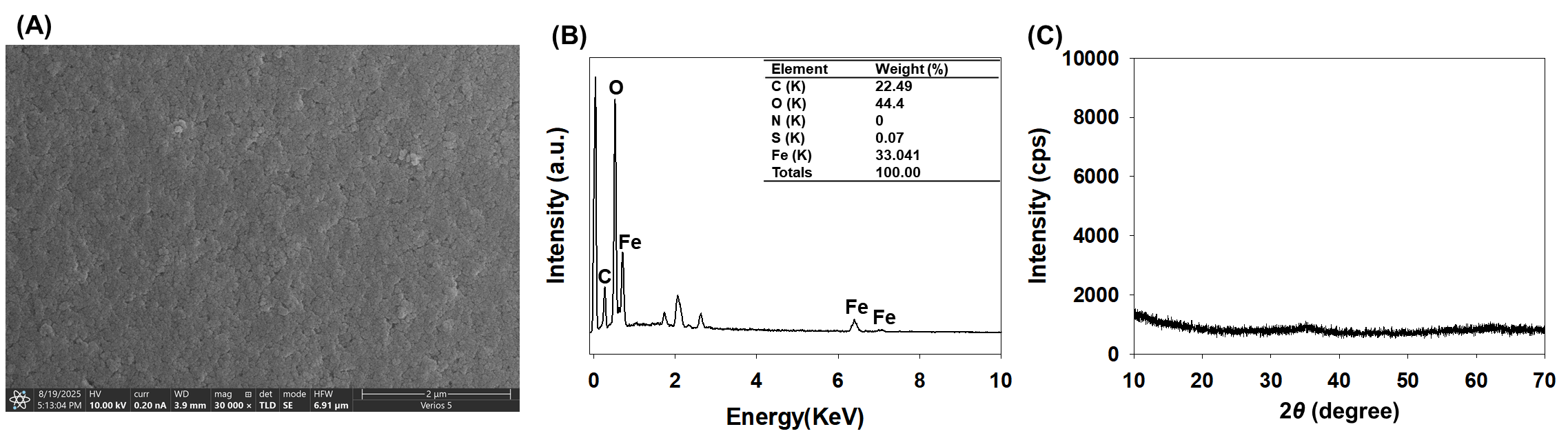
**

**Figure S1.** SEM image (A), EDS spectrum (B), and XRD spectrum (C) of ferrihydrite.

**
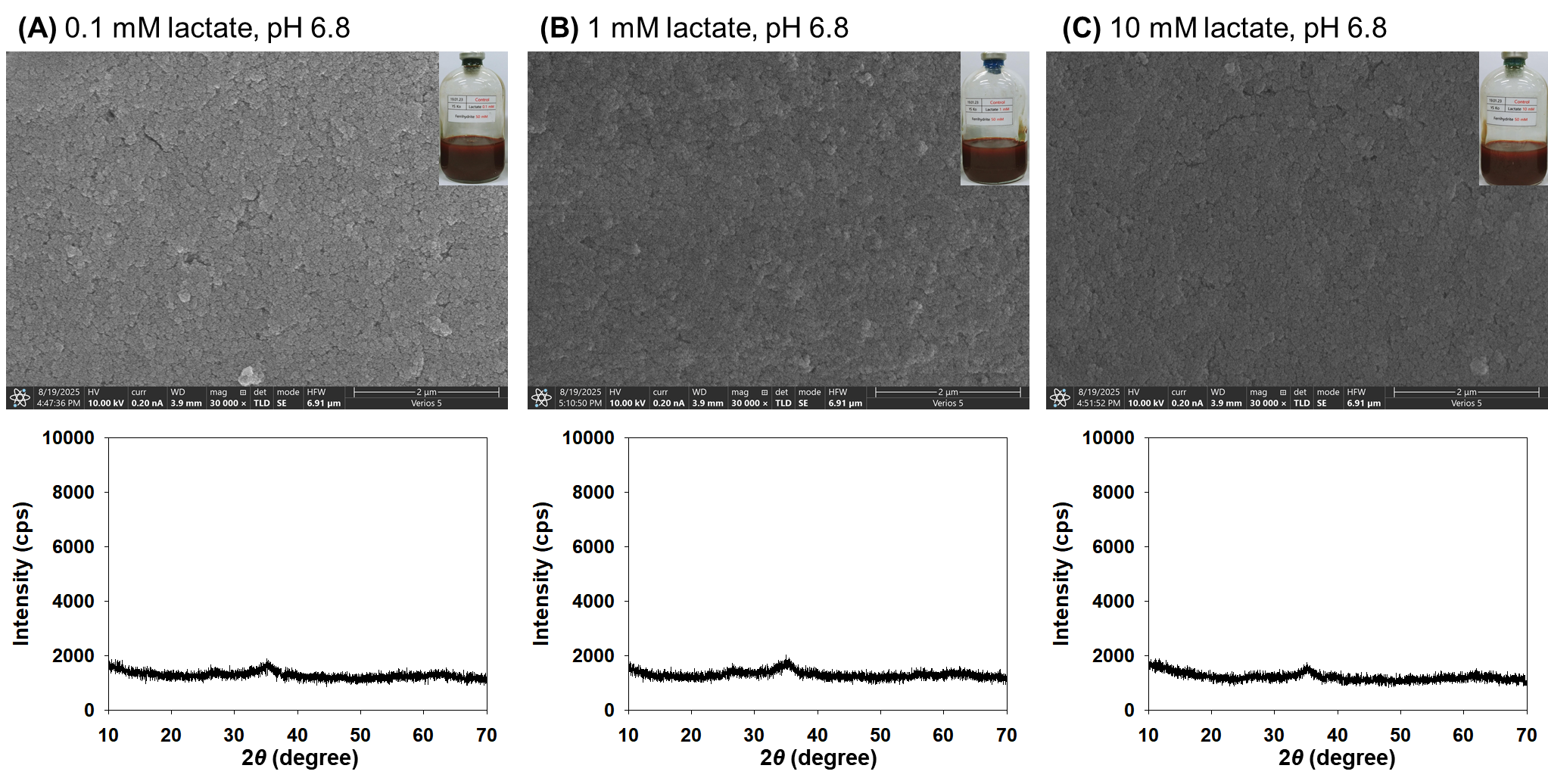
**

**Figure S2.** SEM images and XRD spectra of ferrihydrite in the absence of Shewanella sp. HN-41 at pH 6.8 under different lactate concentrations: 0.1 mM (A), 1 mM (B), and 10 mM (C).


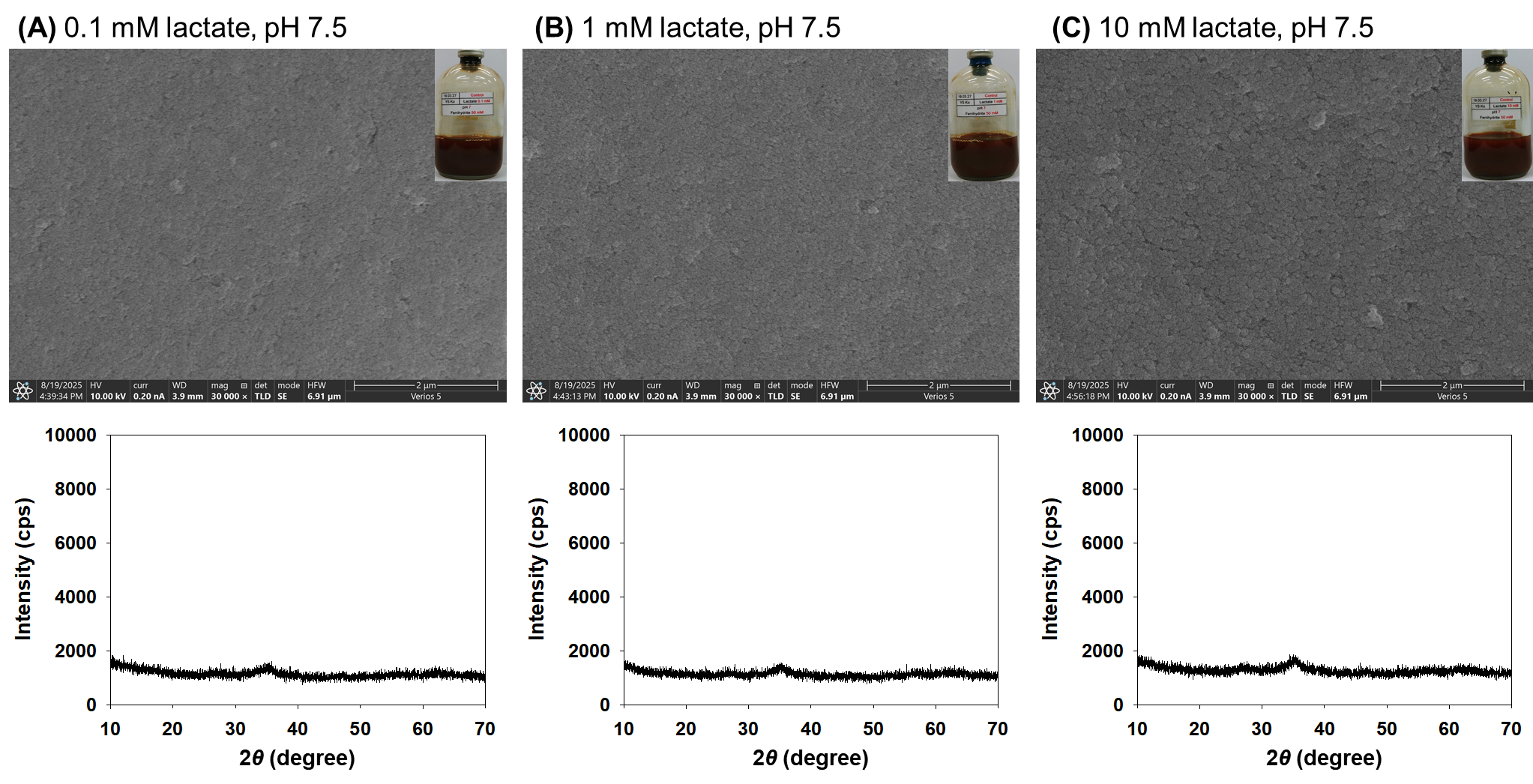


**Figure S3.** SEM images and XRD spectra of ferrihydrite in the absence of *Shewanella* sp. HN-41 at pH 7.5 under different lactate concentrations: 0.1 mM (A), 1 mM (B), and 10 mM (C).
